# Optimization of Non-Uniform Deformation on Piezoelectric Circular Diaphragm Energy Harvester with a Ring-Shaped Ceramic Disk

**DOI:** 10.3390/mi11110963

**Published:** 2020-10-28

**Authors:** Chaoqun Xu, Yuanbo Li, Tongqing Yang

**Affiliations:** Key Laboratory of Advanced Civil Engineering Materials, Ministry of Education, Functional Materials Research Laboratory, School of Materials Science and Engineering, Tongji University, 4800 Cao’an Road, Shanghai 201804, China; 1830608@tongji.edu.cn (C.X.); liyuanbo@tongji.edu.cn (Y.L.)

**Keywords:** piezoelectric energy harvesting, piezoelectric circular diaphragm (PCD), non-uniform strain distribution

## Abstract

Piezoelectric energy harvesting technology using the piezoelectric circular diaphragm (PCD) has drawn much attention because it has great application potential in replacing chemical batteries to power microelectronic devices. In this article, we have found a non-uniform strain distribution inside the PCD energy harvester. From the edge to the center of the ceramic disk, its output voltage first increases and then decreases. This uneven output voltage reduces the output power of the PCD energy harvester. Based on this phenomenon, we reduce the ceramic disk diameter and dig a hole in the center, analyzing the effect of removing the ceramic disk’s low output voltage part on the PCD energy harvester. The experimental results show that removing the ceramic disk’s low output voltage part can improve the output power, reduce the resonance frequency, and increase the optimal impedance of the PCD energy harvester. Under the conditions of 10 g proof mass, 9.8 m/s^2^ acceleration, the PCD energy harvester with a 19-mm diameter and a 6-mm hole can reach a maximum output power of 8.34 mW.

## 1. Introduction

In recent years, with the continuous progress of micro-machining, integrated circuits, and micro-electromechanical systems (MEMS), electronic devices’ design and manufacture are moving towards miniaturization, integration, and networking [1,2,3,4]. On the one hand, electronic devices’ power consumption has continued to decrease and even reach the micro-watt level [5]. On the other hand, the internet of things (IoT) and wireless sensor networks (WSNs) technology are developing rapidly [6]. The demand for microelectronic devices will increase geometrically. At present, the energy supply of microelectronic devices is mainly based on chemical batteries. Still, chemical batteries have many problems that need to be solved, such as large size, limited service life, replaced periodically, produce pollution to the environment, and unsuitability for using in the harsh environment [7]. It has become a problem that urgently needs to be solved to further develop and apply microelectronic devices. Therefore, a continuous, safe, and clean energy supply method is required to replace traditional chemical batteries to power microelectronic devices.

Mechanical vibration energy (human motion, sound waves, machine operation, etc.) exists widely in nature [8,9]. It is not easily affected by the external environment and has a high energy density, which is the focus of the current environmental energy harvesting technology research [10]. Based on the different conversion methods, vibration energy harvesting methods can be divided into electromagnetic conversion [11], electrostatic conversion [12], and piezoelectric conversion [13,14]. Compared with electromagnetic and electrostatic ones, piezoelectric energy harvesting technology has many advantages: simple structure, stable output, large output power density, and excellent integration with micro-electromechanical systems (MEMS) [15,16,17]. It can replace traditional chemical batteries and achieve energy self-sufficiency of microelectronic devices. Previous research studies have reported using piezoelectric energy harvesting to converting ambient mechanical energy into electrical energy [18,19,20,21]. Among the existent piezoelectric components utilized for piezoelectric energy harvesting, piezoelectric circular diaphragm (PCD) energy harvesters have drawn much attention in recent decades [22,23,24]. Compared with the cantilever beam structure energy harvester, the piezoelectric circular diaphragm (PCD) energy harvester can be regarded as multiple fan-shaped cantilever beams connected in parallel, so it has higher output power. Therefore, we choose the PCD energy harvester for experimental research.

For circular piezoelectric energy harvester, Pondrom has proposed a theoretical model of energy harvesting in 2014 [25]. The output power equation is shown as Equation (1):

(1) $$P_{\mathrm{out}}=\frac{m_{s}{}^{2}d_{33}^{2}a^{2}R_{l}\omega^{2}}{\left[{\left(\frac{\omega^{2}}{\omega_{0}^{2}}-1\right)}^{2}+4\zeta_{m}^{2}{\left(\frac{\omega }{\omega_{0}}\right)}^{2}\right]\left[1+{\left(R_{l}C\omega \right)}^{2}\right]}$$

where *m_s_* is the weight of the proof mass, *a* is the acceleration of the system, *R_l_* is the load resistance, *ζ_m_* is the mechanical damping ratio of thickness direction, and *C* is the capacitance of the piezoelectric element. *ω* and *ω_0_* are operating and resonance angular frequency of the energy harvesting system, respectively. *ω_0_* can be calculated by Equation (2):

(2) $$\omega_{0}=\sqrt{\frac{Y_{t}A}{tm_{s}}}$$

where *Y_t_* and A are Young’s Modulus and area of the piezoelectric element, respectively. When the load resistance *R_l_* is equal to the optimal load resistance *R_opt_*, which can be expressed by:

(3) $$R_{l}=R_{opt}=\frac{1}{\omega_{0}C}$$

The maximum output power *P_m_* can be obtained from:

(4) $$P_{m}=\frac{m_{s}{}^{2}d_{33}^{2}a^{2}\omega_{0}}{8\zeta_{m}^{2}C}$$

Equation (4) theoretically explains the factors that affect the PCD energy harvester’s output power, but the actual factors are much more complex. In our previous work, we have found that the PCD energy harvester’s output power is proportional to the piezoelectric material’s coefficient *d*_33_ [26]. Besides, we also analyzed the holding radius of the mass, the bonding radius of the transmission shaft [27], the mass configurations (central proof mass and annular proof mass) [28], and the notched brass substrate on the PCD energy harvester’s output power [29]. Abaqus finite element simulation results show that the piezoelectric ceramic disk’s strain distribution is highly uneven: a large strain field in the inner region and a small strain field in the outer region [30]. This non-uniform strain field distribution results in an uneven output voltage, the inner high output voltage part will charge the outer low output voltage part, causing the overall output voltage to drop, and ultimately the PCD energy harvester’s energy harvesting efficiency is lower than expected. In this article, we designed experiments to demonstrate the phenomenon of the PCD energy harvester’s uneven output voltage. We introduce a PCD energy harvester with a ring-shaped ceramic disk, this design can reduce the difference in output voltage between the outer and inner regions, which leads to a much more homogeneous output voltage distribution of the piezoelectric ceramic disk. Therefore, removing the piezoelectric ceramic disk’s lower output part can improve the PCD energy harvester’s output power.

## 2. Materials and Methods

**Figure 1:**
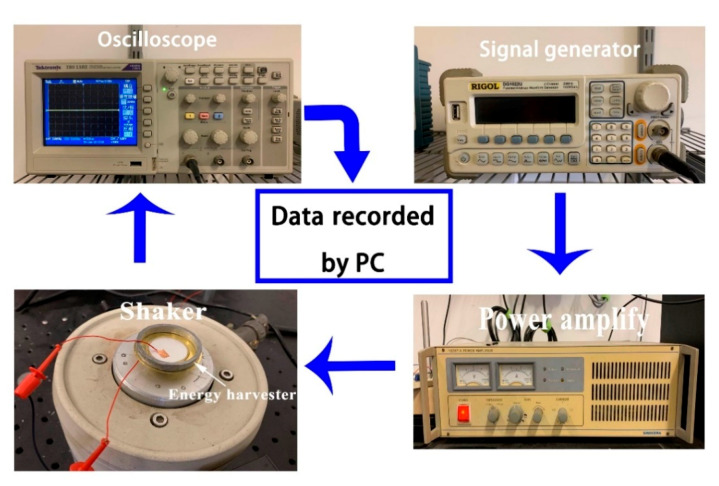
Schematic diagram of the piezoelectric energy harvesting test platform.

The test platform used in this experiment is shown in Figure 1, it is the same as our previous work [31]. The signal generator (33220A, Agilent) controls vibration frequency and amplitude by emitting a vibration signal. The vibration signal is amplified by the power amplifier (YE5871A) and transmitted to the mechanical shaker (JCK-5), which is capable of providing the vibration acceleration. The shaker will vibrate at the corresponding frequency. The PCD energy harvester’s transmission shaft is screwed on the shaker, so the PCD energy harvester vibrates with the shaker and generates electrical energy. A digital oscilloscope (Agilent 3320A) records the real-time voltages of the resistance. In addition, the acceleration amplitude has been calibrated by feedback from a single-axis MEMS accelerometer (BW14100) before the experiment, which is installed with the shaker to obtain the real-time detection of the vibration. Finally, a custom LabVIEW program is set up to control the performance of devices and to collect all generated data. The acceleration of the shaker is fixed to 9.8 m/s^2^ in all experiments.

**Figure 2:**
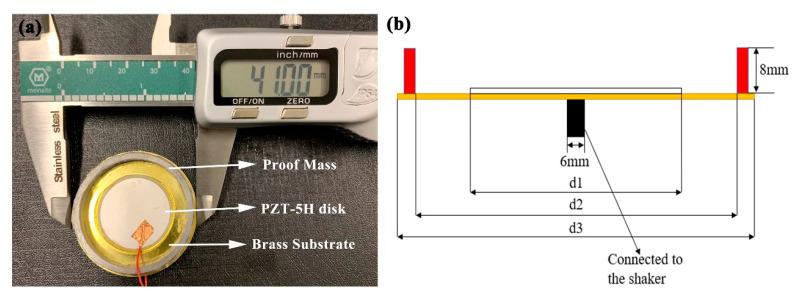
Photograph (**a**) and structural parameters (**b**) of the piezoelectric circular diaphragm (PCD) energy harvester.

The PCD energy harvester adopted in this paper is shown in Figure 2. It is composed of a PZT-5H piezoelectric ceramic disk, a brass substrate, and an annular proof mass. Table 1 shows their specific parameters. The PZT-5H piezoelectric ceramic disk and the brass substrate are connected with epoxy glue. The annular proof mass is stick on the brass substrate with instant glue. A 6-mm transmission shaft is bonded on the brass substrate’s center. The piezoelectric disk we used has a very thin thickness (0.1 mm). During the process of vibration, its thickness direction will deform greatly. Therefore, the main vibration modes of piezoelectric ceramic disks are thickness mode.

## 3. Results and Discussion

**Figure 3:**
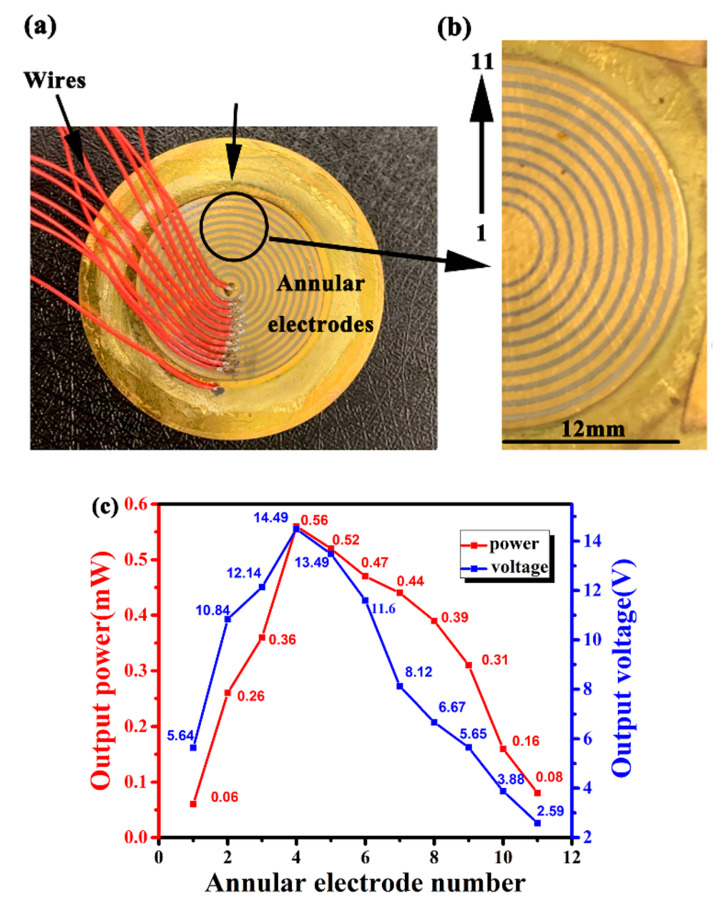
Piezoelectric ceramic disk with annular electrodes (**a**), its partially enlarged details (**b**), and output power and voltage of each electrode (**c**).

In order to analyze the output power of the PCD energy harvester’s different positions, we use an ion sputtering equipment to prepare multiple homocentric annular electrodes on the piezoelectric ceramic disk, as shown in Figure 3a,b. The innermost electrode is numbered as 1 and its diameter is 3 mm. The outermost electrode is numbered as 11 and its diameter is 24 mm. The gap between each electrode is 0.5 mm. Under the conditions of optimal impedance and resonant frequency, each electrode’s output voltage is measured by the digital oscilloscope, then the output power of each electrode is calculated by the Equation (5):

(5) $$P=\frac{U^{2}}{R}$$

where *U* and R are the output voltage and optimal impedance of each electrode, respectively.

The experiment results are shown in Figure 3c. The output power and voltage of the annular electrode 1 are 0.06 mW and 5.64 V, respectively. Then both of the two values raise continuously to 0.56 mW and 14.49 V until it comes to electrode number 4, which is located at the bonding border between the transmission shaft and the copper substrate, where the effective deformation is the largest, so its output power is the highest. After that, the power and voltage drop to 0.08 mW and 2.59 V. From this experiment, we can conclude that the PCD energy harvester’s output charge is uneven. The reason we think that the piezoelectric ceramic disk’s edge part is stick on the proof mass, and it is also far from the transmission shaft, so its output power is low. The piezoelectric ceramic disk’s central part is stick on the transmission shaft, and its deformation is suppressed, so its output power is low. The piezoelectric ceramic disk’s non-uniform deformation results in an uneven output voltage, it is the main problem existing in the PCD energy harvester.

**Figure 4:**
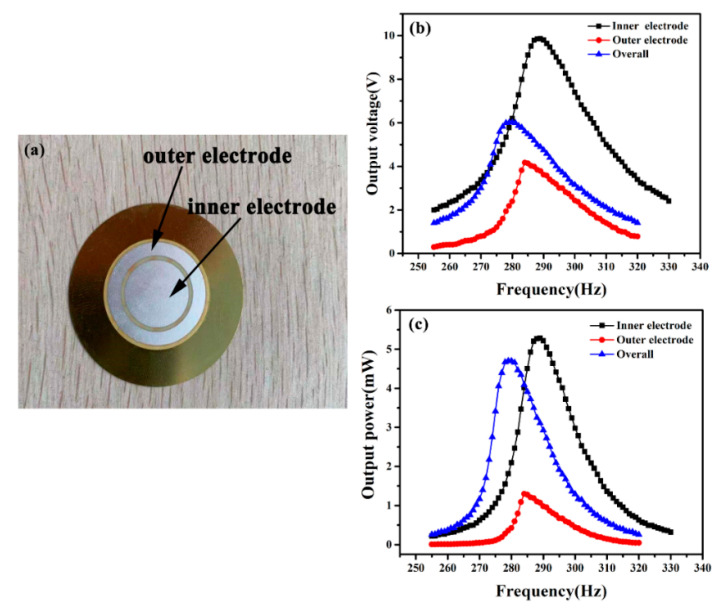
Schematic diagram (**a**), output voltage (**b**), and output power (**c**) of the inner and outer electrodes.

To further analyze the effect of the low output voltage part on the PCD energy harvester, we divided the 25-mm diameter piezoelectric ceramic disk into two parts. Its structure is shown in Figure 4a, the outer electrode diameter is 24 mm and the inner is 15 mm. There is a 1-mm gap separating the outer and inner electrode. The output voltage and power of the inner and outer electrodes are tested, respectively. The results are shown in Figure 4b,c.

The maximum output voltage and power of the inner electrode are 9.86 V and 5.28 mW, and the outer electrode is 4.17 V and 1.29 mW. While the two parts are connected in parallel, the maximum output voltage and power are 6.02 V and 4.64 mW. The output power obtained by connecting two parts in parallel is less than the inner electrode. We think that the inner electrode is in the center of the ceramic disk, which is directly connected to the transmission shaft, and its deformation is large, so its output voltage and power are larger. While the outer electrode is far from the transmission shaft and clamped by the annular proof mass, its deformation is low, so its output voltage and power are lower. When the two parts are connected in parallel, the inner electrode with a higher voltage will charge the outer electrode with a lower voltage, causing the overall output voltage drop. Ultimately the overall output power is less than the inner electrode. This experiment illustrates that the piezoelectric ceramic disk’s low output part is unfavorable for the PCD energy harvester’s output power, which is also the main reason that limits its output power to increase.

**Figure 5:**
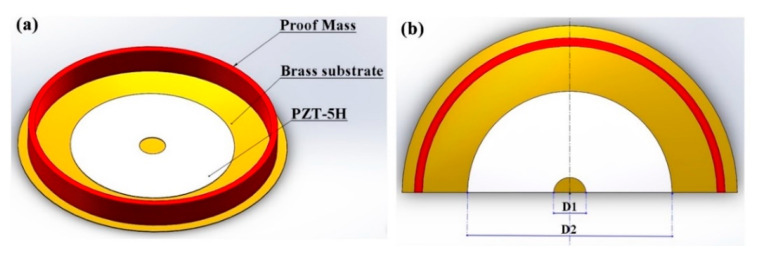
Schematic structure (**a**) and structural parameters (**b**) of the PCD energy harvester with a hole-digging piezoelectric ceramic disk.

The piezoelectric ceramic disk’s low deformation part will reduce the PCD energy harvester’s output power. We remove the edge and the center of the piezoelectric ceramic disk, and the structure is shown in Figure 5. We change the piezoelectric ceramic disk diameter D2 to 25, 22, and 19 mm, and under the same D2, set the digging hole diameter D1 as 0, 2, 4, 6, and 8 mm. They are tested under the conditions of matched impedance.

**Figure 6:**
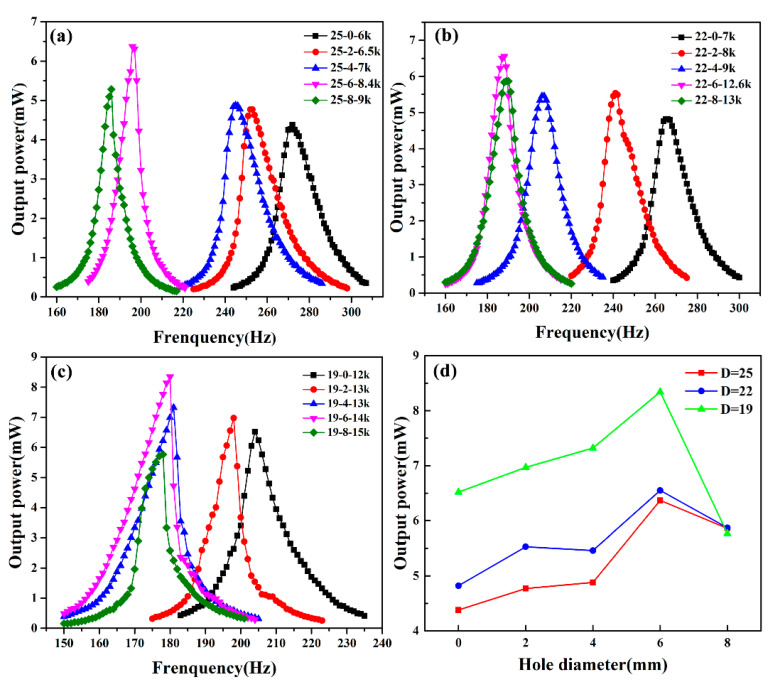
Energy harvesting curve of the 25-mm (**a**), 22-mm (**b**), 19-mm (**c**) hole-digging PCD energy harvesters, and (**d**) their output power comparison diagram.

The output results of 25-mm holed PCD energy harvesters are shown in Figure 6a. The output power of the 25-mm piezoelectric ceramic disk is 4.48 mW at the resonant frequency of 275 Hz. As the hole’s diameter increases, its output power rises continuously until the hole’s diameter is 6 mm and the output power reaches a maximum of 6.37 mW at the resonant frequency of 198 Hz. While at the hole’s diameter of 8 mm, the output power drops to 5.85 mW at the resonant frequency of 180 Hz. From the view of optimal impedance, the optimal impedance increases with the increase of hole diameter. Digging holes reduces the area of the surface electrode, which results in an increase of the optimal impedance. Then we remove the edge part with lower output power and reduce the piezoelectric ceramic disk diameter to 22 mm, the output power results are shown in Figure 6b. Its output power results are similar to 25-mm hole-digging PCD energy harvesters. While the hole diameter increases, the output power first increases and then decreases, the resonance frequency decreases, and the optimal impedance increases. At the hole diameter of 6 mm, the output power reaches a maximum of 6.55 mW at the resonant frequency of 186 Hz. After that, we reduced the piezoelectric ceramic disk diameter to 19 mm, and the output results are shown in Figure 6c. When the hole diameter is 6 mm, the maximum output power reaches 8.34 mW at the resonant frequency of 180 Hz. However, judging from the output power waveform, the output power waveform of the 19-mm hole-digging PCD energy harvesters is relatively sharp, especially when the hole diameter increases to 8 mm, and its output power waveform is very unstable. We think that compared with the 41-mm brass substrate, the area of the ceramic disk is too small, so the PCD energy harvester is already in an unstable state. Digging holes removes the lower output power part, decreases the equivalent stiffness, and reduces the area of the piezoelectric ceramic disk, so it can improve the output power, reduce the resonance frequency, and increase the optimal impedance of the PCD energy harvester. Figure 6d is the output power comparison diagram of 19-, 22-, and 25-mm hole-digging PCD energy harvesters. With the hole diameter increases, the output power first increases and then decreases. When the hole’s diameter is the same as the transmission shaft (6 mm), the output power reaches the maximum. When the diameter of the hole is further increased to 8 mm, the center large output voltage part of the ceramic disk is also removed, thus resulting in a decrease of the output power. Compared with the 25- and 22-mm hole-digging PCD energy harvesters’ output power, the 19-mm hole-digging PCD energy harvester’s is the largest. It also indicates that removing the piezoelectric ceramic disk’s small deformation can improve its output power. When the piezoelectric ceramic disk diameter is 19 mm and the hole-digging size is 6 mm, its output power reaches a maximum of 8.34 mW, which is 86.2% higher than that of a 25-mm no-hole-digging PCD energy harvester.

The performance of some published piezoelectric energy harvesters is listed and compared in Table 2. Compared with other cantilever piezoelectric energy harvesters, our PCD energy harvester can be regarded as multiple fan-shaped cantilever beams connected in parallel, so it has higher output power. The output power (8.34 mW) and output power density (5.73 mW/cm^3^) of our device are obviously higher than other published works. However, compared with other published works, our device has a larger volume and works at the higher resonant frequency (180 Hz), so our normalized power density (31.85 μW·g^−2^·Hz^−1^cm^−3^) is not high enough.

## 4. Conclusions

In summary, this paper demonstrates a non-uniform strain distribution inside the PCD energy harvester, which causes the unevenness of the output voltage. From the edge to the center of the piezoelectric ceramic disk, its output voltage first increases and then decreases, when it is located near the border of the bonding interface, its output voltage reaches maximum. The inner high output voltage part will charge the outer low output voltage part, which causes the overall output voltage to drop, and ultimately the PCD energy harvester’s energy harvesting efficiency is lower than expected. The center and the edge with a lower output voltage will reduce the PCD energy harvester’s output power. When the piezoelectric ceramic disk’s lower output voltage part is removed, the difference in output voltage between the outer and inner regions is thus decreased, which leads to a much more homogeneous output voltage distribution. Therefore, the PCD energy harvester’s output power can be improved. For a 19-mm piezoelectric ceramic disk with a 6-mm hole, the maximum output power and the output power density of the PCD energy harvester at 180 Hz arrived at 8.34 mW, and 5.73 mW/cm^3^, respectively. However, reducing the area of the piezoelectric ceramic disk makes the energy output power waveform become sharper. When reducing the ceramic disk’s area too much (19-mm diameter with 8-mm hole), the structure will be in an unstable state.

## Figures and Tables

**Table 1:** Size parameters of the PCD energy harvester’s each part.

Part	Parameter 1	Parameter 2
PZT-5H	Diameter (d1)/25 mm	Thickness/0.1 mm
Brass substrate	Diameter (d3)/41 mm	Thickness/0.1 mm
Proof mass	Clamping diameter (d2)/38 mm	Weight/10 g

**Table 2:** The performance comparison of the PCD energy harvester with other published works.

Reference	Material/Configuration	Acceleration (g = 9.8 m/s^2^)	Frequency (Hz)	Output Power (Peak Value)	Power Density (mW/cm^3^)	Normalized Power Density (μW·g^−2^·Hz^−1^cm^−3^)
Janphuang et al. [32] 2014	Thinned bulk PZT	1 g	96	82.4 μW	1.73	17.95
Singh et al. [33] 2015	PZT with SSHI circuit	4.6 g	90	385 μW	3.27	1.72
Song et al. [34] 2017	PZT with spiral shape	0.25 g	68	0.023 μW	0.21	49.17
Kim et al. [35] 2010	Bulk PZT	0.255 g	109.5	0.53 mW	0.57	80.05
Zou et al. [36] 2017	PZT with magnet	0.4 g	9.9	194 μW	0.485	303.12
Tang et al. [37] 2018	Thinned bulk PZT	1.5 g	34	226.6 μW	1.71	22.22
This work	Bulk PZT	1 g	180	8.34 mW	5.73	31.85

## References

[1] Fang H.-B., Liu J.-Q., Xu Z.-Y., Dong L., Wang L., Chen D., Cai B.-C., Liu Y. (2006). Fabrication and performance of MEMS-based piezoelectric power generator for vibration energy harvesting. Microelectron. J..

[2] Lin S.-C., Wu W.-J. (2013). Fabrication of PZT MEMS energy harvester based on silicon and stainless-steel substrates utilizing an aerosol deposition method. J. micromech. Microeng..

[3] Liu H., Lee C., Kobayashi T., Tay C.J., Quan C. (2012). A new S-shaped MEMS PZT cantilever for energy harvesting from low frequency vibrations below 30 Hz. Microsyst. Technol..

[4] Jiang L., Yang Y., Chen R., Lu G., Li R., Li D., Humayun M.S., Shung K.K., Zhu J., Chen Y. (2019). Flexible piezoelectric ultrasonic energy harvester array for bio-implantable wireless generator. Nano Energy.

[5] Beeby S.P., Tudor M.J., White N.M. (2006). Energy harvesting vibration sources for microsystems applications. Meas. Sci. Technol..

[6] Roundy S., Wright P.K., Rabaey J. (2003). A study of low level vibrations as a power source for wireless sensor nodes. Comput. Commun..

[7] Ruffo R., Hong S.S., Chan C.K., Huggins R.A., Cui Y. (2009). Impedance Analysis of Silicon Nanowire Lithium Ion Battery Anodes. J. Phys. Chem. C.

[8] Invernizzi F., Dulio S., Patrini M., Guizzetti G., Mustarelli P. (2016). Energy harvesting from human motion: Materials and techniques. Chem. Soc. Rev..

[9] Gardonio P., Zilletti M. (2016). Vibration energy harvesting from an array of flexible stalks exposed to airflow: A theoretical study. Smart Mater. Struct..

[10] Roundy S. (2005). On the Effectiveness of Vibration-based Energy Harvesting. J. Intell. Mater. Syst. Struct..

[11] Wang P., Dai X., Fang D.-M., Zhao X.-L. (2007). Design, fabrication and performance of a new vibration-based electromagnetic micro power generator. Microelectron. J..

[12] Altena G., Renaud M., Elfrink R., Goedbloed M.H., De Nooijer C., Van Schaijk R. (2013). Design improvements for an electret-based MEMS vibrational electrostatic energy harvester. J. Phys. Conf. Ser..

[13] Howells C.A. (2009). Piezoelectric energy harvesting. Energy Convers. Manag..

[14] Kim S., Clark W.W., Wang Q.-M. (2005). Piezoelectric Energy Harvesting with a Clamped Circular Plate: Experimental Study. J. Intell. Mater. Syst. Struct..

[15] Kim H.S., Kim J.-H., Kim J. (2011). A review of piezoelectric energy harvesting based on vibration. Int. J. Precis. Eng. Manuf..

[16] Toprak A., Tigli O. (2014). Piezoelectric energy harvesting: State-of-the-art and challenges. Appl. Phys. Rev..

[17] Yang Z., Zhou S., Zu J., Inman D. (2018). High-Performance Piezoelectric Energy Harvesters and Their Applications. Joule.

[18] Wu J., Shi H., Zhao T., Yu Y., Dong S. (2016). High-Temperature BiScO_3_-PbTiO_3_Piezoelectric Vibration Energy Harvester. Adv. Funct. Mater..

[19] Homayouni-Amlashi A., Mohand-Ousaid A., Rakotondrabe M. (2020). Analytical Modelling and Optimization of a Piezoelectric Cantilever Energy Harvester with In-Span Attachment. Micromachines.

[20] Wang W., Yang T., Chen X., Yao X. (2012). Vibration energy harvesting using a piezoelectric circular diaphragm array. IEEE Trans. Ultrason. Ferroelectr. Freq. Control..

[21] Tian W., Ling Z., Yu W., Shi J. (2018). A Review of MEMS Scale Piezoelectric Energy Harvester. Appl. Sci..

[22] Qi L. (2019). Modeling of functionally graded circular energy harvesters due to flexoelectricity. Appl. Math. Model..

[23] Xiao Z., Yang T.Q., Dong Y., Wang X.C. (2014). Energy harvester array using piezoelectric circular diaphragm for broadband vibration. Appl. Phys. Lett..

[24] Yuan T.-C., Yang J., Chen L.-Q. (2018). Nonlinear dynamics of a circular piezoelectric plate for vibratory energy harvesting. Commun. Nonlinear Sci. Numer. Simul..

[25] Hillenbrand J., Hillenbrand J., Sessler G.M., Bös J., Melz T. (2014). Vibration-based energy harvesting with stacked piezoelectrets. Appl. Phys. Lett..

[26] Dong Y., Yang T., Xiao Z., Liu Y., Wang X. (2015). Performance enhancement of PZT material for circular diaphragm energy harvester. J. Mater. Sci. Mater. Electron..

[27] Yang Y., Wang S., Stein P., Xu B.-X., Yang T. (2017). Vibration-based energy harvesting with a clamped piezoelectric circular diaphragm: Analysis and identification of optimal structural parameters. Smart Mater. Struct..

[28] Shu F., Yang T., Liu Y. (2018). Enhancement of power output by a new stress-applied mode on circular piezoelectric energy harvester. AIP Adv..

[29] Han Y., Li Y., Yang Y., Li M., Yang T. (2020). Improvement of uneven charge distribution on piezoelectric circular diaphragm with notched-substrate. AIP Adv..

[30] Yang Y., Li Y., Guo Y., Xu B.-X., Yang T. (2018). Improved vibration-based energy harvesting by annular mass configuration of piezoelectric circular diaphragms. Smart Mater. Struct..

[31] Liu Y., Yang T., Shu F. (2016). Optimization of energy harvesting based on the uniform deformation of piezoelectric ceramic. Funct. Mater. Lett..

[32] Janphuang P., Lockhart R., Uffer N., Briand D., De Rooij N.F. (2014). Vibrational piezoelectric energy harvesters based on thinned bulk PZT sheets fabricated at the wafer level. Sens. Actuators A Phys..

[33] Singh K.A., Kumar R., Weber R.J. (2015). A Broadband Bistable Piezoelectric Energy Harvester with Nonlinear High-Power Extraction. IEEE Trans. Power Electron..

[34] Song H.-C., Kumar P., Maurya D., Kang M.-G., Reynolds W.T., Jeong D.-Y., Kang C.-Y., Priya S. (2017). Ultra-Low Resonant Piezoelectric MEMS Energy Harvester with High Power Density. J. Microelectromech. Syst..

[35] Kim M., Hoegen M., Dugundji J., Wardle B.L. (2010). Modeling and experimental verification of proof mass effects on vibration energy harvester performance. Smart Mater. Struct..

[36] Zou H.-X., Zhang W.-M., Li W.-B., Hu K.-M., Wei K.-X., Peng Z., Meng G. (2017). A broadband compressive-mode vibration energy harvester enhanced by magnetic force intervention approach. Appl. Phys. Lett..

[37] Tian Y., Li G., Yi Z., Liu J., Yang B. (2018). A low-frequency MEMS piezoelectric energy harvester with a rectangular hole based on bulk PZT film. J. Phys. Chem. Solids.

